# Studying missingness in spinal cord injury data: challenges and impact of data imputation

**DOI:** 10.1186/s12874-023-02125-x

**Published:** 2024-01-06

**Authors:** Lucie Bourguignon, Louis P. Lukas, James D. Guest, Fred H. Geisler, Vanessa Noonan, Armin Curt, Sarah C. Brüningk, Catherine R. Jutzeler

**Affiliations:** 1 Department of Health Sciences and Technology (D-HEST), ETH Zurich, Universitätstrasse 2, 8092 Zürich, Switzerland; 2 Schulthess Klinik, Lengghalde 2, 8008 Zürich, Switzerland; 3 Neurological Surgery and the Miami Project to Cure Paralysis, U Miami, Miami, FL 33136 USA; 4 Department of Medical Imaging, College of Medicine, University of Saskatchewan, Saskatoon, Saskatchewan Canada; 5 Praxis Spinal Cord Institute, Vancouver, British Columbia Canada; 6 Spinal Cord Injury Center, University Hospital Balgrist, University of Zurich, Lengghalde 2, 8006 Zürich, Switzerland; 7 SIB Swiss Institute of Bioinformatics, Lausanne, Switzerland

**Keywords:** Missing data, Imputation, Spinal cord injury, Simulation study

## Abstract

**Background:**

In the last decades, medical research fields studying rare conditions such as spinal cord injury (SCI) have made extensive efforts to collect large-scale data. However, most analysis methods rely on complete data. This is particularly troublesome when studying clinical data as they are prone to missingness. Often, researchers mitigate this problem by removing patients with missing data from the analyses. Less commonly, imputation methods to infer likely values are applied.

**Objective:**

Our objective was to study how handling missing data influences the results reported, taking the example of SCI registries. We aimed to raise awareness on the effects of missing data and provide guidelines to be applied for future research projects, in SCI research and beyond.

**Methods:**

Using the Sygen clinical trial data (*n* = 797), we analyzed the impact of the type of variable in which data is missing, the pattern according to which data is missing, and the imputation strategy (e.g. mean imputation, last observation carried forward, multiple imputation).

**Results:**

Our simulations show that mean imputation may lead to results strongly deviating from the underlying expected results. For repeated measures missing at late stages (> = 6 months after injury in this simulation study), carrying the last observation forward seems the preferable option for the imputation. This simulation study could show that a one-size-fit-all imputation strategy falls short in SCI data sets.

**Conclusions:**

Data-tailored imputation strategies are required (e.g., characterisation of the missingness pattern, last observation carried forward for repeated measures evolving to a plateau over time). Therefore, systematically reporting the extent, kind and decisions made regarding missing data will be essential to improve the interpretation, transparency, and reproducibility of the research presented.

**Supplementary Information:**

The online version contains supplementary material available at 10.1186/s12874-023-02125-x.

## Introduction

In the era of big data, medical research fields are facing a data challenge. The surge of new mathematical and statistical methods promises to help understand the progression of patients’ recovery following a medical event, improve diagnosis and prognosis, thereby enhancing patients’ care. However, such models require sufficient data, preferable in the magnitude of thousands of entries, to identify recurring patterns and infer prediction rules. In a number of medical fields, such as the ones studying rare conditions (e.g., spinal cord injury [SCI]) or rehabilitation, the sample size available is typically smaller and further limited by the presence of missing data, with only a fraction of the overall data being available. With its low prevalence and particular recovery pattern (i.e., time of onset precisely defined followed by recovery which plateaus between six to 12 months after initial event), traumatic SCI constitutes an ideal study case for missing data, which can be transferred to other medical fields. The last few decades saw the emergence of SCI datasets, such as the European Multicenter Study about Spinal Cord Injury (EMSCI) [1] or National Spinal Cord Injury Model Systems [2], including over 5000 and 50,000 patients, respectively, partially filling the gap of data availability. However, these registries, like most medical data, are prone to missing entries (e.g., patients lost to follow-up, incomplete data entry, injury conditions making it impossible to perform certain tests, different medication schemes etc.).

According to Rubin [3], missing data is categorized into three patterns, missing completely at random (MCAR), missing at random (MAR) and missing not at random (MNAR) (see Table 1). More precisely, MCAR refers to values, which are missing not only independently of their true unknown value, but also of the value of the other variables present in the data. In other words, data MCAR are equivalent to sampling a representative subset of the complete population. When data is MAR, a missing entry is not directly related to the underlying value, but related to other variables collected along with the variable in which missing data is observed, i.e., the proportion of missing entries differs between identifiable subgroups in the data. Finally, data are MNAR when the underlying missing value is directly related to the entry being missing. Previous studies have shown that MNAR could lead to biased interpretation of the results of statistical analysis [4–7]. Bias is defined as a deviation from the truth (e.g., either over- or underestimating an effect) which can lead to erroneous conclusions [8]. This phenomenon is important when dealing with medical data, as they are prone to data MAR and MNAR [9, 10].

**Table 1:** Missing data patterns. LEMS: lower extremity motor score

Pattern	Definition	Example
Missingness completely at random	Values are missing independently of their true unknown value and independently of other variables	A LEMS value is missing for participant A with no underlying reason
Missingness at random	Values are missing independently of their true unknown value but the pattern depends on other variables	A LEMS value is missing for participant A because they had a cast at the time of assessment, i.e. knowing the cast status gives information on whether LEMS value will be missing or not
Missingness not at random	Having a value missing depends on the true unknown value	A LEMS value is missing for participant A because their injury was so severe that they could not come for the assessment, i.e. the underlying true LEMS gives information on whether LEMS value will be missing or not

Independent of the missing data pattern, incomplete reports often lead to the exclusion of patients as most mathematical models require so called “complete data”, effectively performing complete case analysis (CCA). This does not only represent a missed opportunity to benefit from the entire sample available, but can also lead to conclusions that are not representative of the entire population, and/or transferable to other populations. Despite those limitations, CCA is the most frequent strategy applied when handling missing data in SCI registries, although the resulting limitations are not always explicitly acknowledged [11–13]. It has been shown that this strategy, when applied to other medical research questions, could introduce bias in the results reported [14, 15]. Beyond performing a complete case analysis, there exist multiple ways of handling missing data. Imputation, in particular, refers to the procedure of inferring likely values of the missing entries [16]. These strategies can be categorized into single or multiple imputation, which would infer one or multiple likely value(s), respectively. Likewise, imputation methods can consider only one variable (e.g., mean imputation) or multiple variables at a time (e.g., model-based imputation such as predictive mean matching [pmm]). Previous studies have reported better performances of multiple imputation compared to single imputation strategies when data was missing in a HIV cohort [17] or in oncogene expression profiles [18]. Those results are in line with the underlying motivation for multiple imputation. Having multiple plausible imputed values allows to take into account the uncertainty when estimating missing values. On the other hand, single imputation might impute falsely precise values [16].

A particularity of traumatic SCI disease progression is that patients to some extent recover with time. Most of the recovery takes place in the first 6 months after injury followed by a plateau between six and 12 months after injury [19]. The recovery is characterized by non-linear and highly heterogeneous recovery patterns. Owing to a scarcity of studies, the effect of missing data and imputation is not well understood for SCI datasets. Importantly, other medical scenarios involving repeated measures may show a similar plateau in the evolution of variables over time (e.g., observational studies characterising recovery in rehabilitation centers following stroke [20] or traumatic brain injury [21], partial recovery following relapses in multiple sclerosis [22]).

To address this knowledge gap, we designed a simulation study characterizing the impact of three key parameters on the results reported, namely the variable in which data is missing, the pattern of missingness, and finally the imputation strategy applied. Firstly, considering the recovery pattern following SCI, we hypothesized that performing an imputation by last observation carried forward (LOCF) for the outcome variable evaluated at week 52 would not significantly affect the models’ outcomes. However, we expected that carrying an observation from earlier time points (e.g., 16 weeks post injury) would introduce bias in the interpretation of a model owing to the non-linearity of the recovery trajectory. Secondly, we suspected that, while CCA is an efficient and unbiased way of handling missing data when it is MCAR, it would introduce bias when data is MAR or MNAR in the field of SCI as well. When data is MAR or MNAR, we hypothesized that multiple imputation strategies, which consider the uncertainty in the imputation process, would outperform ad-hoc and single imputation strategies. Finally, we hypothesized that mean imputation is not a suitable strategy to handle missing SCI data, regardless of the missingness pattern, since the assumption of normally distributed data is not met for many SCI-related outcomes, such as the lower extremity motor score (LEMS).

Overall, our study evaluates extensively the impact of missingness on the analysis of medical data, taking the example of SCI. Using data from the Sygen clinical trial, a well established SCI data source, provides an opportunity to reconsider the importance of missing data when studying SCI data and beyond.

## Methods

### Data source

#### Sygen cohort

The Sygen project was a multicenter, randomized, double-blinded clinical trial conducted between 1992 and 1998 in the United States, to evaluate the effect of GM-1 ganglioside on recovery following acute SCI [23–25]. Failing to demonstrate superiority over placebo in terms of recovery following SCI, the Sygen study has emerged as a valuable data source for research projects owing to the diligent data collection and the size of the cohort, which is considerably larger than many contemporary cohorts [26]. All enrolled patients were treated with methylprednisolone sodium succinate (MPSS) according to the NASCIS II protocol as part of the standard of care [27]. The design of this clinical trial included the assessment of neurological status at predefined time points. A baseline measurement (before 72 hours from injury and after the competition of the NASCIS II [28]), 4, 8, 16, 26, and 52 weeks following injury. The delayed baseline exam was centered around 48 hours after injury. This time delay in baseline exam allowed a complete neurological examination, also considering any recovery from hemodynamic normalization occurring between the emergency room and 48 hours after injury. Among other variables, neurological level of injury (NLI), motor scores (lower extremity [LEMS] and upper extremity [UEMS] motor scores), sensory scores (pin prick and light touch) [29] and the American Spinal Injury Association (ASIA) Impairment Scale (AIS) [30] were reported. Overall, the cohort includes 797 participants, with a majority of severe injuries (AIS A, 56%).

### Simulation study

**Fig. 1:**
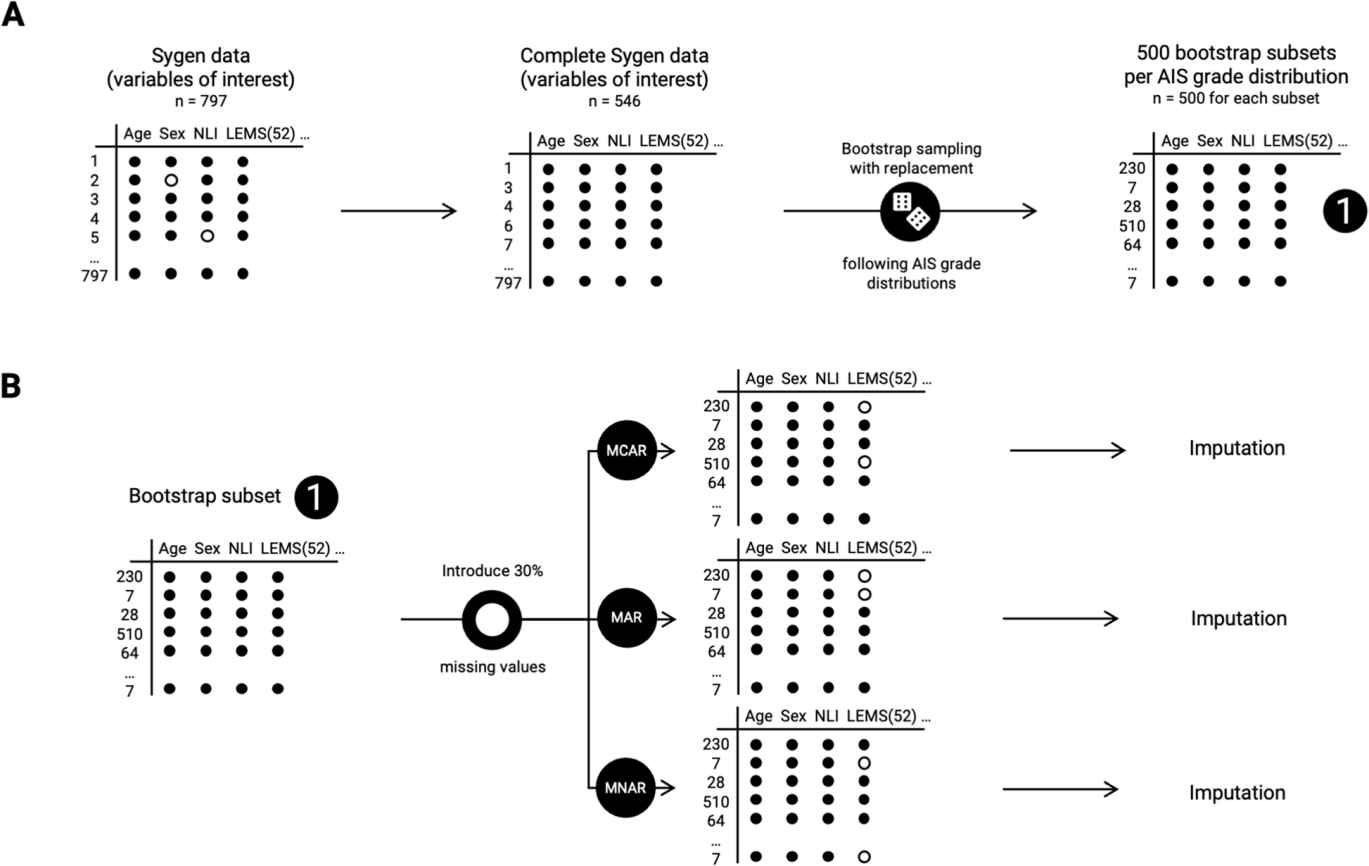
Simulation study overview. **A** The first step leads to the definition of 500 bootstrap subsets, with *n* = 500 in each subset; **B** In each bootstrap subset, 30% missing data is introduced in the variable for lower extremity motor score (LEMS) (either at baseline or at recovery) according to one of the three patterns of missingness (missing completely at random [MCAR], missing at random [MAR], missing not at random [MNAR]), independently, before being imputed. Empty circles represent missing entries, while plain circles represent known values; AIS: American spinal injury association impairment scale

We conducted a simulation study where missing values were artificially introduced in data otherwise complete. We assessed three key characteristics of the missing data: the type of variable in which data is missing (i.e. outcome versus explanatory variable), the patterns of missingness and the imputation strategy. We summarized the simulation study in Fig. 1.

#### Definition of the bootstrap subsets

We first selected all patients, who had data for LEMS at delayed baseline exam (referred to as “baseline”) stage and chronic/52 weeks stage (referred to as “chronic”), as well as AIS grade, NLI, sex and age at baseline. To emulate a plausible research hypothesis, we considered the following model:

1 $$LEM{S}_{chronic}\sim LEM{S}_{baseline}+ AIS\ \mathit{\operatorname{grad}}{e}_{baseline}+ NLI+ age+ sex$$

where we intended to study the association between LEMS at the chronic stage (outcome variable) and LEMS at baseline (explanatory variable), taking into account potential confounders, such as the AIS grade, NLI, age, and sex at baseline. Note, for simplicity LEMS scores were considered to be continuous scores and NLI a binary variable, taking either the value “cervical” or “thoracic”. Patients with lower injuries (i.e., at and below L1) were excluded in the original study [23].

In order to assess how variable the effects of missing data and data imputation are, we performed a bootstrap sampling with replacement to create 500 bootstrap subsets (*n* = 500 entries for each) with fixed AIS grade distributions (Fig. 1A). The distributions followed either the original AIS grade distribution from the complete Sygen data for the variables of interest, or with balanced AIS grade groups (25% of grade A, B, C and D in the final cohorts, AIS grade E was not included as this category is not present in the original study [23]).

#### Introduction of missing data

In each bootstrap subset, we introduced 30% missing values in two of the variables, namely LEMS at chronic stage and LEMS at baseline (Fig. 1B). This percentage was chosen based on the percentage of missing data observed in the Sygen cohort (24.1%) and was set to a higher, more conservative percentage. In this study, we focused on simulations in which missing data would be introduced in one variable at a time, whilst the rest of the variables would be complete, as a way to simplify the task at hand. The choice of these variables was motivated by their different status in the example model (outcome and explanatory, respectively).

Missing values were introduced according to three patterns: MCAR, MAR, and MNAR as described in Rubin in 1976 [3]. For modeling MCAR, if LEMS at baseline is missing for a specific patient, the missing value would be unrelated to all other variables, including the outcome variable LEMS at chronic stage (i.e., 52 weeks after injury). As such, disregarding those entries should not introduce bias, provided a sufficient sample size [31]. In the case of values MAR, information about the missing value can be retrieved by studying the other variables. To simulate this behavior values MAR were introduced depending on the variable sex, where being male made it twice more likely to have a value missing, compared to being female. Finally, MNAR is a pattern, in which the unknown true value influences whether the value is missing or not. In this study, we simulated that patients with less severe injuries would be more likely to be missing. Specifically, high LEMS (i.e. above the LEMS 30th percentile) were four times more likely to be missing compared to low LEMS (i.e. below the LEMS 30th percentile). The four times difference reflects the four AIS grade categories (from A to D), closely related to the LEMS [32]. The 30th percentile threshold was chosen to match the 30% missing data introduced, easily allowing for a change in percentage of missing data introduced in future studies.

#### Imputation strategies

The introduced missing values were imputed with three types of procedures: ad-hoc methods, single imputations and multiple imputations.

Ad-hoc methods included mean imputation [33] and last observation carried forward (LOCF) [34]. The latter was used for imputation of the outcome variable only (LEMS at chronic stage), where missing data were replaced by LEMS assessed 26 weeks after injury. Intending to test the time sensitivity of the LOCF, we repeated the analysis using LEMS at 26 weeks as the primary outcome variable, and imputed it using LEMS available at week 16. We hypothesized, based on the recovery profile following SCI [19], that LOCF from week 26 to chronic stage would be more relevant than LOCF from week 16 to week 26, where a substantial amount of recovery is still likely to occur. We focused our analyses on outcomes measured at week 26 and week 52 after initial injury as they are the reference timepoints used in clinical trials to assess recovery following SCI [23, 35].

Single imputation consisted of three main steps: (1) taking the set of patients, which are not missing for a defined variable; (2) fitting a model to describe this variable according to all others; and (3) predict likely values for the missing ones, based on the fitted model. For example, if one imputes missing LEMS score at baseline, the fitted model would be:

2 $$LEM{S}_{baseline}\sim AIS\ \mathit{\operatorname{grad}}{e}_{baseline}+ NLI+ age+ sex$$

Note that we excluded the outcome variable as it represents information that is not available at baseline. Different models can be used to fit the data available for imputation. In this simulation study, we focused on linear regression (LR) [36], k-nearest neighbors (k-NN) [37], and support vector machines (SVM) [38] using two types of kernel (linear and radial basis function [RBF]) and random forest (RF) [39], as they represent a commonly used set of machine learning models for prediction tasks [40]. All models included a 5-fold cross-validation scheme for hyperparameter optimization. The corresponding parameter grids can be found in Additional file 1.

Single imputation is inherently limited as it does not provide uncertainty related to the imputed value. Multiple imputation addresses this challenge: the imputation is performed multiple times (25 times here, as a compromise between increased power and reasonable run time [41]) before being pooled. Similarly as for single imputation, the outcome variable was excluded from the imputation of the explanatory variable. Models including predictive mean matching (pmm), linear regression imputation (norm.predict) and tree-based method (random forest, rf) were chosen, as implemented in the R *mice* package [42]. Models for multiple imputation were chosen to match the models used in the single imputation with the aim to increase the comparability between the two approaches.

Based on our study design, we chose to pool the data before fitting the example model featured in Eq. (1), with the final imputed value being the mean of all imputed values for the LEMS continuous variables [43]. This approach was taken in order to obtain a single imputed value for each missing entry to allow for the computation of metrics (see *Metrics*). However, it does not match the flow advocated in the implementation for multiple imputation as presented in the R *mice* package. In order to ensure that this change in procedure does not impair the outcome of the multiple imputation, we compared both approaches in the Additional file 2.

Finally, we performed a CCA, where any patient (case) with at least one missing value among the variables described in the section *Definition of the bootstrap subsets* would be disregarded and the analysis performed solely using patients for whom the entire set of variables was observed.

All imputation strategies were compared to their corresponding bootstrap subset when complete, designated as baseline subset.

### Evaluation of data imputation

Following imputation, we sought to evaluate and compare the different imputation strategies tested. We employed various methods to both examine population- (i.e., statistical tests, beta coefficient comparisons) and individual-level (i.e., metrics) performance of imputation methods in restoring the missing entries.

#### Statistical tests

##### Two-sample Kolmogorov-Smirnov test

We tested the null hypothesis considering that the two sets of observations were drawn from the same unknown probability distribution, using a two-sample Kolmogorov-Smirnov test [44], as implemented by the ks.test function in the *stats* R package. The two sets of observations considered were either the variable before and after introducing the missing values, or the variable before introducing the missing values compared with the variable after imputation, or the variable after introducing missing values and the variable after imputation. Note that in a typical imputation situation, true values are not available, and thus only comparison between the set of non-missing values and the set of values after imputation would be possible.

##### Chi-squared goodness of fit test

The chi-squared goodness of fit test, chisq.test in R, was employed to compare the proportion of categorical variables between two cohorts. Its null hypothesis states that the sample to be tested follows the hypothesized distribution from the other cohort.

##### Little’s test

Little’s test was first described in 1988 [45]. It tests the null hypothesis that data is missing completely at random in a given cohort. In our framework, the mcar_test function, implemented in the *naniar* R package [46], first allowed us to ensure that the missing data was introduced as intended, i.e., MCAR or not (Section 3.1.2), and was further used to describe the missingness in the original Sygen cohort (Section 3.3).

##### Metrics

We used the following metrics for a quantitative comparison of variables, continuous and categorical, in their complete version versus after imputation. All imputation methods were subsequently ranked to determine, for each metric, which imputation method would consistently lead to imputations closer to the true values across repeated runs.

##### Mean absolute error (MAE)

The MAE computes the average absolute difference between a known true value  and its corresponding imputed version  for all  entries  for which missingness was introduced:

3 $$MAE = \frac{1}{n} \sum_{i}^{n} |y_{i} - \hat{y}_{i}|$$

MAE is a negatively-oriented score, which means lower values indicate better imputation performance. This metric has the advantage of being intuitively interpretable as it is expressed in the units of the variables, i.e., a MAE of 3.5 for LEMS at baseline would mean that, on average, the imputed values for LEMS missing at baseline are 3.5 points away from their true values.

##### Root mean squared error (RMSE)

The RMSE differs from the MAE as it squares the difference between true and imputed values, thus penalizing large errors more. By taking the square root of the overall average of differences, it allows one to interpret the RMSE on the scale of the initial values, similarly to the MAE. Likewise, a RMSE of 0 corresponds to the best possible imputation.

4 $$RMSE = \sqrt{\frac{1}{n} \sum_{i}^{n} (y_{i} - \hat{y}_{i})^{2}}$$

#### Comparison of beta coefficients after linear regression (LR) using imputed data

The last method we employed to assess the quality and impact of imputation was to fit a linear regression (LR) based on the simulated research question stated in Eq. (1) and compare the beta coefficients for the explanatory variables estimated from a LR based on the complete set of data and the imputed data. This method allowed us to highlight the difference in the conclusion drawn from a research question according to its study design regarding the way to handle missing data. We considered the 95% confidence interval (CI) and mean difference in beta coefficients for each explanatory variable (i.e., LEMS at baseline). For an imputation method to be considered unbiased, the CI should include the value 0 (i.e., it is likely that the true difference between the beta coefficients is negligible) and be as small as possible.

For all tests, the threshold of *p* < 0.05 was considered significant and led to rejecting the corresponding null hypothesis. Analyses were performed with R Statistical Software (version 3.6.0) and Python (version 3.7.4).

## Results

### Description of the data

#### Full cohort and selected complete case cohort from the Sygen trial

Summary statistics of the variables of interest for our simulation study are presented in Table 2. After including only complete cases for the variables of interest, the cohort was reduced from 797 to 546 patients. Comparing the two cohorts did not yield significant differences in terms of the proportion of sex (chi-squared test, X-squared = 0.66, df = 1, *p*-value = 0.42), age (two-sample Kolmogorov-Smirnov test, D = 0.02, *p*-value = 0.99), level of injury (chi-squared test, X-squared = 0.25, df = 1, *p*-value = 0.62) or LEMS at baseline and at recovery (two-sample Kolomogorov-Smirnov test, D = 0.01, *p*-value = 1, for both variables). When comparing the proportions of AIS grades and considering missing data as a category in itself, which would not be present by design in the cohort with only complete data, a significant difference is reported between the two cohorts (chi-squared test, X-squared = 73.96, df = 4, *p*-value < 0.001). Since this difference is likely to be driven by the additional missing category, we performed the same test using only the actual grades available. It revealed no significant difference in the proportions of each grade between the two cohorts (X-squared = 0.84, df = 3, *p*-value = 0.84).

**Table 2:** Characteristics of the Sygen cohort for the variables of interest, before and after selecting for complete cases

		Entire cohort	Complete cases only	*p*-value
Number of patients	n	797	546	
Sex				0.42
	n (% male)	643 (80.7)	433 (79.3)	
	NA, n (%)	0 (0.0)	0 (0.0)	
Age				1
	mean (SD)	32.5 (13.4)	32.00 (13.3)	
	NA, n (%)	0 (0.0)	0 (0.0)	
LEMS at week 01				1
	mean (SD)	2.7 (7.2)	2.7 (7.1)	
	median [Q1-Q3]	0 [0–0]	0 [0–0]	
	NA, n (%)	74 (9.3)	0 (0.0)	
LEMS at week 26				1
	mean (SD)	12.1 (18.7)	11.9 (18.9)	
	median [Q1-Q3]	0 [0–29]	0 [0–28]	
	NA, n (%)	168 (21.1)	27 (4.9)	
LEMS at week 52				1
	mean (SD)	12.8 (19.3)	12.6 (19.3)	
	median [Q1-Q3]	0 [0–32]	0 [0–31]	
	NA, n (%)	192 (24.1)	0 (0.0)	
Level of injury				0.62
	Cervical, n (%)	600 (75.3)	406 (74.4)	
	Thoracic, n (%)	197 (24.7)	140 (25.6)	
AIS grade				0.84
	A, n (%)	446 (56.0)	356 (65.2)	
	B, n (%)	77 (9.7)	59 (10.8)	
	C, n (%)	149 (18.7)	108 (19.8)	
	D, n (%)	31 (3.9)	23 (4.2)	
	NA, n (%)	94 (11.8)	0 (0.0)	< 0.001

*LEMS* lower extremity motor score, *AIS* American Spinal Injury Association Impairment Scale, *NA* not available (missing data), *SD* standard deviation, *Q1* first quartile, *Q3* third quartile; continuous variables (LEMS and age) were compared using a two-sample Kolmogorov-Smirnov test, categorical variables (sex, level of injury and AIS grade) were compared using a chi-squared goodness of fit test

#### Subsets from the cohort of complete cases

Variables of interest are summarized for every AIS grade distribution in Table 3. Each value is reported as the mean of the variable’s values across the 500 subsets drawn according to the same AIS grade distribution as in the cohort with complete cases from the Sygen data, or with balanced AIS grade groups.

**Table 3:** Characteristics of the 500 bootstrap subsets (500 entries each) created according to the AIS grade distributions present in the Sygen cohort including only complete cases for the variables of interest, and a balanced cohort, where all 4 grades are present in equal proportions

Outcome at week 52		Sygen complete cases (subsets)	Balanced (subsets)
Number of patients	n	500	500
Number of male	mean (SD)	396.8 (8.6)	385.5 (9.2)
Age [years]	mean (SD)	32.0 (13.3)	34.2 (14.0)
LEMS at week 01	mean (SD)	2.7 (7.0)	8.5 (12.1)
	median [95% CI]	0 [0–0]	0 [0–0]
LEMS at week 26	mean (SD)	11.9 (18.8)	25.6 (21.5)
	median [95% CI]	0 [0–0]	34 [33–34]
	NA, n	24.9 (5.2)	22.8 (4.6)
LEMS at week 52	mean (SD)	12.6 (19.3)	26.2 (21.7)
	median [95% CI]	0 [0–0]	35 [35–36]
Level of injury	cervical, mean (SD)	372.3 (9.8)	412.7 (8.4)
	thoracic, mean (SD)	128.7 (9.8)	87.3 (8.4)
AIS grade	A, n (%)	325 (65.0)	125 (25.0)
	B, n (%)	55 (11.0)	125 (25.0)
	C, n (%)	100 (20.0)	125 (25.0)
	D, n (%)	20 (4.0)	125 (25.0)

*LEMS* lower extremity motor score, *AIS* American Spinal Injury Association Impairment Scale, *SD* standard deviation, *CI* confidence interval

In order to test whether the missing data were introduced as intended (i.e., following MCAR, MAR and MNAR patterns, respectively), we performed a Little’s test for each subset and for each variable in which missing data was introduced, separately. As expected, the null hypothesis, stating that the data is MCAR, is mostly rejected when missingness is introduced at random or not at random (range: 491–500 subsets out of 500, Additional file 3). When missingness is introduced completely at random, it is expected that the null hypothesis would be rejected in 5% of the 500 subsets since we defined our significance threshold to be less than 0.05. That represents a 5% probability that the null hypothesis, whilst being correct, is rejected. This expectation matches the observation across subsets in which missingness was introduced completely at random, with the null being rejected in 26 (5.2%) and 32 (6.4%) bootstrap subsets, depending on the AIS distribution (Additional file 3). Overall, this step allows us to assume that the missingness patterns were introduced appropriately.

Following the introduction of the missing data, we evaluated the impact of the missing data on the distribution of the variable in which it was introduced. When tested with the two-sided Kolmogorov-Smirnov test, introducing MCAR and MAR did mostly not significantly change the distributions of the two variables (LEMS at baseline and recovery) (Additional file 4). By contrast, introducing MNAR introduced a shift in the distribution of the variables for the majority (500 and 305/500 when AIS grade distribution follows the complete Sygen data’s distribution and a balanced AIS grade distribution, respectively) of the bootstrap subsets. Introducing MNAR in LEMS at recovery in a population where the proportions of AIS grades are balanced (25% for each group), was an exemption to that observation. In this particular case, the null hypothesis of the two-sided Kolmogorov-Smirnov test, stating that the values of LEMS at recovery before and after introducing MNAR were drawn from the same underlying population, was rejected for 305 subsets out of 500. In comparison, it was rejected for all subsets in a similar population AIS grade distribution, when missingness was introduced at random.

### Performance of imputation methods

#### Statistical tests

The results comparing the distributions of the true and imputed values after introducing missing data are summarized in Additional files 5 and 6. While introducing data MCAR or MAR did not lead to significant shifts in distributions (see Section 3.1.2), we observed that the imputation methods introduced shifts irrespective of the underlying AIS grade distribution in the population or the variable with missing entries. Similarly, we noted that across variables, underlying AIS grade distributions in the samples and missingness patterns, the majority or mean imputation systematically shifted the distribution of the imputed variable.

When data was MNAR, the distributions of the resulting population were often significantly different from the initial population (from 305 to 500 out 500 subsets, Section 3.1.2 and Additional file 4). Following imputation, this shift was more likely to be reversed as the underlying population structure approached balanced proportions in AIS grades (e.g., 150 versus 295 subsets out of 500 had a significantly different population distribution after imputation with multiple random forest when data is MNAR in LEMS at baseline, Additional file 5). The imputation method that led to the least number of subsets in which a shift was still observed was imputation using a RF (simple imputation, four subsets when data MNAR, Additional file 5), followed by pmm (multiple imputation, 14 subsets when data MNAR, Additional file 5) for the LEMS at baseline. One exception arose when missingness was introduced in the outcome variables, LEMS at the chronic stage, where imputation with LOCF led to sample distributions that were never significantly different from the true population (Additional file 6). This observation also held true when the outcome variable was measured 26 weeks after injury and imputation was based on data collected 16 weeks after injury (Additional file 7). However, when both LEMS at chronic stage and week 26 were missing, LOCF could not be performed and led to the exclusion of a mean of 6.8 (standard deviation: 2.5), 6.8 (standard deviation: 2.5) and 6.5 (standard deviation: 2.6) entries per bootstrap subset, when LEMS at chronic stage was MCAR, MAR and MNAR, respectively.

#### Metrics

Testing for difference in distributions is equivalent to looking at the performance of the imputation at a population level. It is, however, also interesting to see at the scale of the individual imputed values how the imputation performs. For that purpose, we computed various metrics (Methods) to quantify the agreement between individual imputed values and their true counterpart, across bootstrap subsets.

**Fig. 2:**
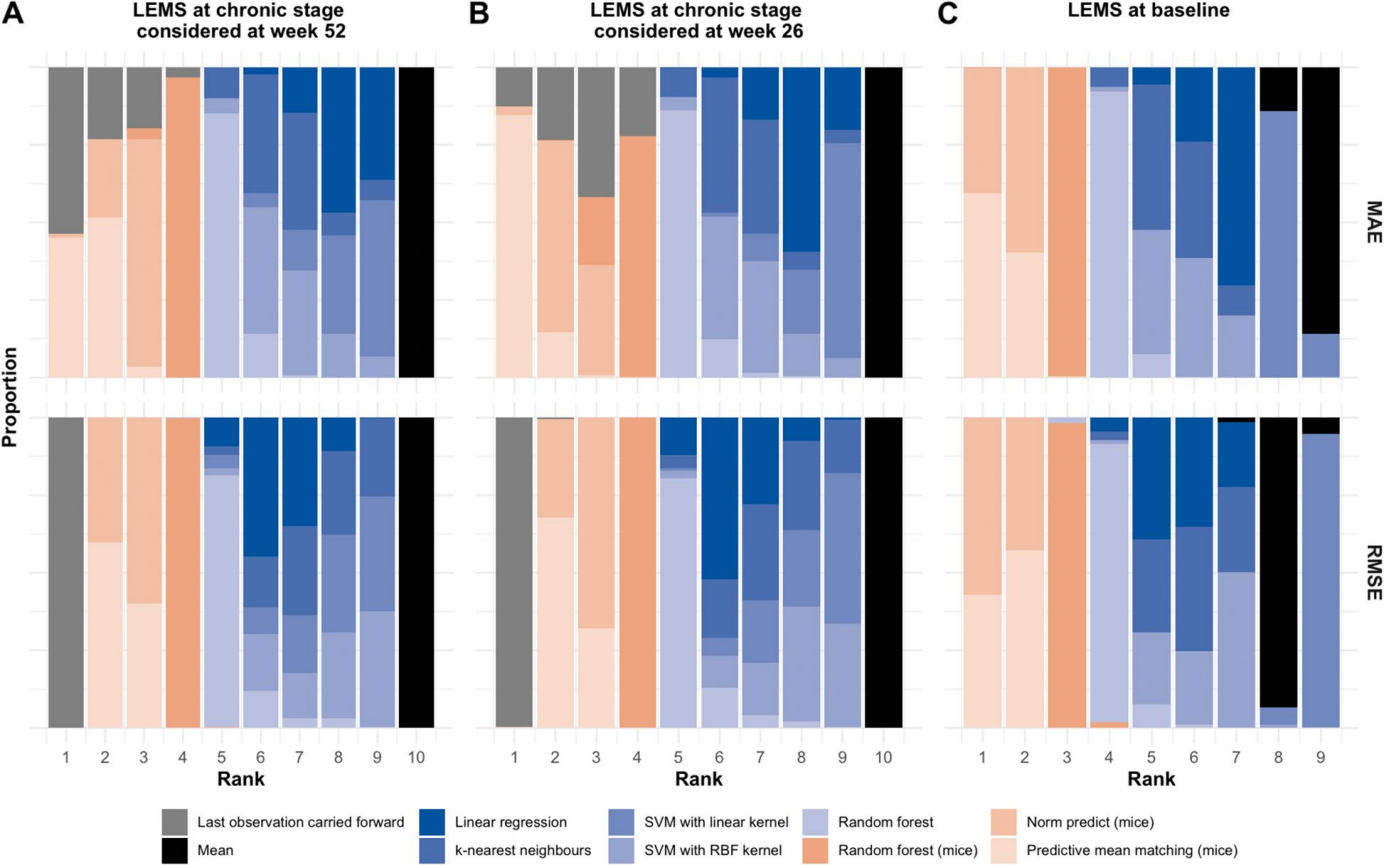
Imputation methods ranked from lowest (1) to highest (9 or 10) metrics’ values when introducing missing data not at random in **A**. LEMS at outcome considered at week 52. **B** LEMS at outcome considered at week 26. **C** LEMS at baseline. For each subset (*n* = 500), missing data is introduced and imputed using all methods. Within each subset, imputation performance is compared between imputation methods and ranked from best performance (i.e., closest to 0 and ranked 1) to lowest performance (i.e., highest metric value and ranked 9 or 10). We display the proportion of subsets (out of 500) per rank and imputation method; LEMS: lower extremity motor score, MAE: mean absolute error, RMSE: root mean squared error, SVM: support vector machines, RBF: radial basis function

Two main observations were similar to the ones obtained when comparing imputation methods at the population level by means of statistical tests. Firstly, LOCF was the imputation method leading to the lowest MAE and RMSE, when imputing the outcome variable evaluated at week 52 (Fig. 2A). When the outcome was measured at week 26 after injury, LOCF was still consistently among the top four imputation methods but was outperformed by pmm (Fig. 2B). Secondly, mean imputation led to the lowest ranked metric values in most of the scenarios, regardless of the other three parameters to be studied in this simulation (i.e., AIS grade distribution, missingness patterns, variables to be imputed, Additional files 8 and 9, Fig. 2). Multiple imputation, on the contrary, was always ranked the highest (following LOCF if present), across all metrics, with a slight advantage to pmm and norm.predict (ranked in the top two, after LOCF, in all the simulations) over multiple RF (ranked third, or fourth when LOCF is present, in over 90% of the simulations), when imputing LEMS variables (Fig. 2). We also observed that the distribution of the metrics values were less variable with multiple imputation when repeating the process in 500 bootstrap subsets compared to the single imputation methods (standard deviation of distribution of MAE when LEMS at chronic stage MAR: 0.97, 0.71, 0.24 and 0.21 when imputed using k-nearest neighbors, linear regression, pmm and norm.predict, respectively, Additional file 9).

#### Comparison of beta coefficients after linear regression (LR) using imputed data

**Fig. 3:**
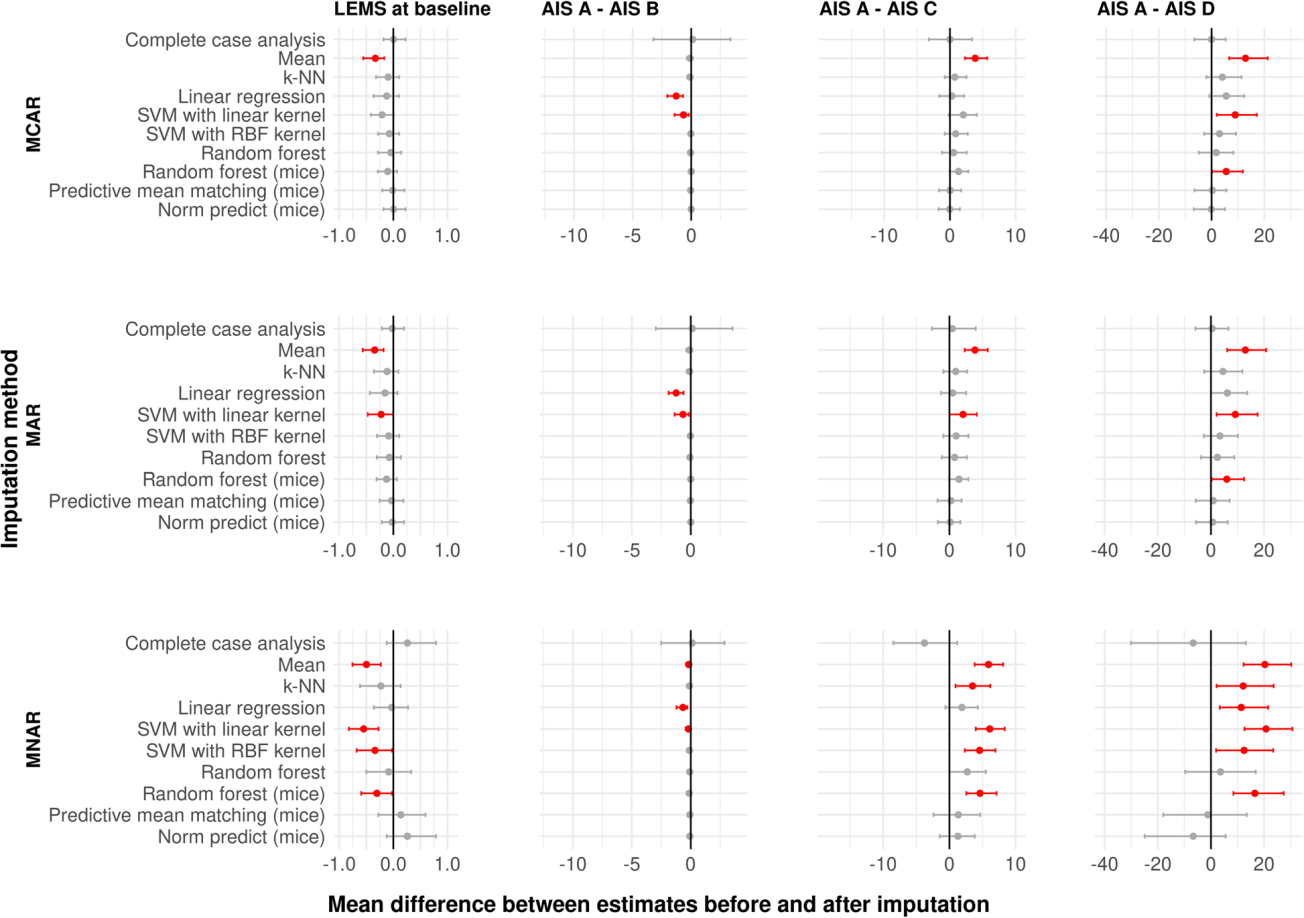
Mean and confidence interval of the difference between estimates from the data before introducing missingness in LEMS at baseline and after imputation. Each row corresponds to missing data being introduced using a different missingness pattern (MCAR, MAR and MNAR from top to bottom). Each column corresponds to the estimate of one explanatory variable (LEMS at baseline, AIS B compared to AIS A, AIS C compared to AIS A and AIS D compared to AIS A, from left to right). Intervals displayed in red do not contain the value 0. MCAR: missing completely at random, MAR: missing at random, MNAR: missing not at random, LEMS: lower extremity motor score, AIS: American Spinal Injury Association Impairment Scale, k-NN: k-nearest neighbors, SVM: support vector machines, RBF: radial basis function

As shown in Fig. 3, mean imputation for LEMS missing at baseline consistently introduced a bias in coefficients estimated via linear regression, with the magnitude of the bias increasing from data MCAR to MAR to MNAR (mean difference between estimates of beta for LEMS at baseline of − 0.33, − 0.35, and − 0.50 when data MCAR, MAR and MNAR, respectively). In contrast, bias would not be introduced when performing a CCA, i.e. zero would also be present in the CI. This imputation method, however, led to wide CIs in the difference between coefficients estimated on the entire data versus on the imputed data (e.g. when estimating the effect of AIS grade D in comparison with AIS grade A, 95% CI of [− 6.5; 5.3], [− 5.8; 6.6] and [− 30.1; 13.1] for data MCAR, MAR and MNAR, respectively). Taken together, Fig. 3 supports the use of multiple imputation methods such as pmm and norm.predict in imputing missing LEMS at baseline, as those methods did not introduce bias and resulted in smaller CI, especially with data MNAR ([− 17.9; 13.5] for estimates of the effect of AIS grade D in comparison with AIS grade A). When imputing missing LEMS at week 52, LOCF produced estimates of beta that were both unbiased and close to the estimates derived from the entire data (Additional file 10). If the outcome is evaluated at week 26, imputation of missing data using LOCF uses information available at week 16. Despite using information from an earlier time in the recovery process, it appears to still be the most reliable imputation method with no bias introduced, except when data is MNAR. In that case, although the estimates repeatedly deviated from the expected ones, the bootstrap CI is tight compared to CI obtained with other imputation methods ([− 2.5; − 0.3] and [− 3.1; − 0.5] for the estimates for AIS B and C versus AIS grade A, respectively, Additional file 11).

### Application of studying missing values to real-world data

**Fig. 4:**
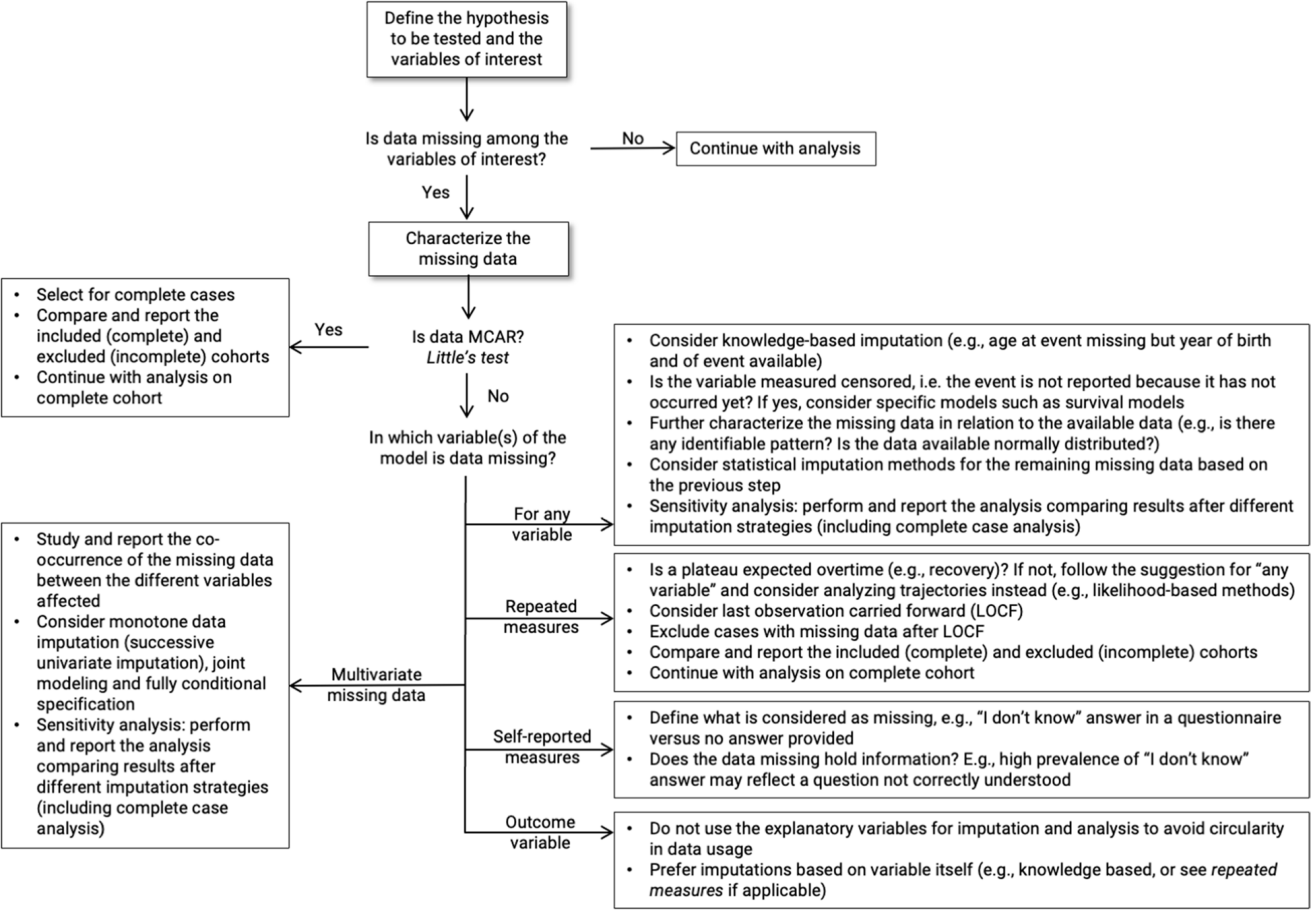
General consideration when facing missingness in medical data

As presented in Table 2, the full Sygen cohort (*n* = 797) presents missing entries for LEMS at both time points and AIS grade at week 1, when taking into account the variables studied in our example model. Sex, age and NLI, however, had no missing data. Additional file 12 illustrates the co-occurrence of missing data across the variables considered. The hypothesis of the data being MCAR was rejected when taking all the variables of the model together (Little’s test, statistic = 76.4, df = 44, number of missing patterns = 8, *p*-value = 0.002). The variable with most missing entries, LEMS evaluated at week 52, presents with 24.1% missing data, making our simulation with 30% missing data more conservative. Notably, both LEMS at week 52 and 26 were missing for 136 (17.1%) participants. It is important to highlight that this subset could not benefit from a reliable imputation based on the LOCF strategy. However, 56 participants (7.0%) could be included in such an analysis by imputing the missing outcome variable using the LOCF strategy. For the participants in which either LEMS or AIS grade at baseline was missing, imputation could be envisaged, preferably through multiple imputation. General consideration on how to apprehend missing data, both based on knowledge from the literature and results from the simulation study described here, are presented in Fig. 4.

## Discussion

In this simulation study, we aimed to address the impact of missing data in SCI data sources on the results reported. We specifically focused on three key components that could affect the analysis itself and the interpretation of the results: the type of variable in which data is missing, the pattern in which the data is missing (i.e., MCAR, MAR, MNAR), and the imputation strategy applied.

In agreement with reports from other medical fields [4, 47, 48], we showed that data MNAR is more likely to lead to biased subsequent analysis as it might change the distribution of the data available for analysis (Additional file 4). Likewise, disregarding the presence of data MNAR by performing an analysis based on complete cases can also lead to erroneous conclusions compared to an analysis that would have been performed on the entire sample with no missing data (Fig. 3) considering the large CI of the difference between the true estimate and the estimate obtained from the imputed data. This point is particularly crucial as most studies currently perform complete case analysis [49–52], and we reported absolute effect sizes greater than 5 (when MNAR and considering AIS D or C compared to A), surpassing the threshold of 5 points considered as clinically significant for LEMS [53]. It could also not be excluded that data was MNAR in the Sygen data (Section 3.3). The latter is likely to hold true in most SCI datasets (EMSCI database [26]) owing to the nature of the data itself (i.e., observational medical data). When dealing with MNAR, CCA did not consistently lead to the introduction of bias in the estimates of beta coefficients, contradicting our initial hypothesis. We observed, however, that multiple imputation strategies, in particular pmm and norm.predict equally led to unbiased estimates but with narrower CIs, suggesting a lower variance in the estimates. Similarly, multiple imputation methods were more likely to generate distributions closer to the initial true distribution. Taken together, it seems that, for this cohort, handling MNAR with multiple imputation would be more appropriate than to use CCA.

While LOCF is only possible in the case of variables being observed at multiple time points and may not be appropriate for other medical outcomes [54, 55], our study supports the use of this imputation for SCI-related outcomes such as the LEMS. We were able to show that performing LOCF from week 26 to week 52 leads to a population similar to the true underlying population in terms of distribution (Additional file 7), individual values imputed (Metrics) and estimated beta coefficients from the LR model (Additional file 9). This is likely attributable to the very characteristic recovery trajectory of SCI, including a plateau starting six to 12 months after the initial injury. Contrary to our initial expectation, this observation still held true when performing LOCF from week 16 to week 26 (Additional file 10). However, it is important to note that this conclusion might be specific to outcomes with this particular recovery trajectory, and might not be transferable to outcomes where no plateau can be observed (both SCI-related or unrelated outcomes). LOCF is also a well suited imputation method for outcome variables as it only relies on data that will be further used at a later stage of the data analysis or modeling process. This effectively prevents introducing circularity, which in turn improves the potential transferability of the reported results to a clinical setting. However, it should be noted that in longitudinal studies, one may take advantage of the repeated measures and analyze the entire recovery trajectory rather than the mere association between baseline and chronic measures. In such cases, likelihood-based methods (e.g., mixed-effects models) would be advantageous. Indeed, they inherently allow for MCAR and MAR, or specification of the joint distribution between the data present and missing data when data is MNAR, thus not requiring imputation [56].

### Limitations

It is important to note that the interpretation of this study might be limited by a few factors. Firstly, we studied imputation for repeated measures in the context of SCI using LOCF considering carrying forward information from week 26 to week 52, and from week 16 to week 26. However, we have not explored whether carrying forward values from earlier timepoints (e.g., week 4 or 8) would lead to equally reliable imputed values. Additionally, the exact time points to consider for a valid LOCF will depend on the spacing between repeated measures available and the expected trajectory and timeframe of the variable of interest. Secondly, we restricted our analysis to a fixed amount of missing data (i.e., 30%). This percentage was chosen based on the actual percentages of missing data observed in the variables studied in the original data used and was fixed to a higher percentage to be more conservative while being able to compare our results across variables. Thirdly, we only investigated continuous variables. Dealing with missing data in categorical variables (e.g., AIS grade, assessing SCI severity) would require the use of other models (e.g., proportional odds logistic regression for multiple imputation) and give rise to specific challenges (e.g., how to impute a category that is not present in the data but theoretically possible). Additionally, we did not consider self-reported variables, which missingness can carry information and should therefore be studied beyond imputation [57, 58]. These points have not been explored as a means to limit the complexity of our primary analysis, but constitute the starting point of future work. Finally, we focused on missing data being present in one variable at a time, i.e., univariate imputation. Investigating the multivariate missing data problem poses additional challenges, including but not restricted to combining different missingness patterns, introducing circularity when imputing outcomes based on explanatory variables, or potentially masking meaningful information from the co-occurrence of missing entries. In such cases, imputation strategies can range from combining multiple univariate imputation (i.e., monotone data imputation), conditional univariate models or modeling the joint distributions within the entire dataset [59]. Similarly, exploring different research questions or at the scale of larger databases was beyond the scope of this initial analysis but would benefit from their own study. Accordingly, it would be interesting to extend this simulation study and further analysis of missing data using additional SCI datasets such as the EMSCI or the Rick Hansen Spinal Cord Injury Registry (RHSCIR) [60], and similar observational datasets beyond SCI such as the Transforming Research and Clinical Knowledge in Traumatic Brain Injury initiative [61] focusing on traumatic brain injury.

### Conclusion

Our study raises awareness regarding the presence and impact of missing data in medical data sources (e.g., clinical trials, registries), taking the example of SCI. We demonstrated that disregarding missing data could not only result in a significant loss of information, but also lead to erroneous conclusions. Hence, we see this work as a first step towards systematically considering and reporting the presence of missing data as part of good practices in SCI data analysis and beyond.

### Supplementary Information

**Additional file 1.**

**Additional file 2.**

**Additional file 3.**

**Additional file 4.**

**Additional file 5.**

**Additional file 6.**

**Additional file 7.**

**Additional file 8.**

**Additional file 9.**

**Additional file 10.**

**Additional file 11.**

**Additional file 12.**

## Data Availability

Anonymized data used in this study is available upon request to the corresponding author and in compliance with the General Data Protection Regulation (EU GDPR). The code describing the analysis can be accessed on our GitHub repository (https://github.com/lbourguignon/missingness-in-SCI-data).

## Abbreviations

SCI: Spinal cord injury
EMSCI: European multicenter study about spinal cord injury
NLI: Neurological level of injury
LEMS: Lower extremity motor score
UEMS: Upper extremity motor score
ASIA: American spinal injury association
AIS: American spinal injury association impairment scale
MCAR: Missing completely at random
MAR: Missing at random
MNAR: Missing not at random
LOCF: Last observation carried forward
CCA: Complete case analysis
LR: Linear regression
k-NN: k-nearest neighbors
SVM: Support vector machines
RBF: Radial basis function
RF: Random forest
pmm: predictive mean matching
polr: proportional odds model
MAE: Mean absolute error
RMSE: Root mean squared error
df: degree of freedom
CI: Confidence interval
