# Supplementary material for: Studying missingness in spinal cord injury data: challenges and impact of data imputation
Source: BMC Med Res Methodol. 2024 Jan 6;24:5. doi: 10.1186/s12874-023-02125-x (PMC10770973; doi:10.1186/s12874-023-02125-x)
Supplement: Supplementary file 1 — Additional file 1. [file 12874_2023_2125_MOESM1_ESM.docx]

**Additional File 1.** Parameter grid of the models used for simple imputation. ovr: one-vs-the-rest; SVM: support vector machines; RBF: radial basis function; oob: out-of-bag; sqrt: square root; mse: mean squared error; mae: mean absolute error

| Model | Hyperparameter | Values |
| --- | --- | --- |
| Logistic regression | | |
|  | scaler | {‘passthrough’, StandardScaler()} |
| Linear regression | | |
|  | scaler | {‘passthrough’, StandardScaler()} |
|  | class_weight | {None, ‘balanced’} |
|  | multi_class | {‘auto’, ‘ovr’, ‘multinomial’} |
|  | penalty | {‘none’} |
|  | max_iter | 50000 |
| K-nearest neighbours | | |
|  | scaler | {‘passthrough’, StandardScaler()} |
|  | n_neighbors | {1, 2, 3, 4, 5, 6, 7, 8, 9, 10} |
|  | weights | {‘uniform’, ‘distance’} |
|  | algorithm | {‘ball_tree’} |
|  | metric | {‘minkowski’} |
|  | p | 2 |
| SVM with linear kernel | | |
|  | scaler | {‘passthrough’, StandardScaler()} |
|  | C | {10^-5^, 10^-4^, 10^-3^, 10^-2^, 10^-1^, 10^0^, 10^1^, 10^2^, 10^3^} |
|  | kernel | {‘linear’} |
|  | max_iter | 50000 |
| *when used for classification* | *class_weight* | *{None, ‘balanced’}* |
| SVM with RBF kernel | | |
|  | scaler | {‘passthrough’, StandardScaler()} |
|  | C | {10^-5^, 10^-4^, 10^-3^, 10^-2^, 10^-1^, 10^0^, 10^1^, 10^2^, 10^3^} |
|  | kernel | {‘rbf’} |
|  | max_iter | 50000 |
| *when used for classification* | *class_weight* | *{None, ‘balanced’}* |
| Random forest | | |
|  | scaler | {‘passthrough’, StandardScaler()} |
|  | bootstrap | {True} |
|  | oob_score | {True, False} |
|  | n_estimators | {15, 25, 50, 75, 100} |
|  | max_features | {‘auto’, ‘sqrt’, ‘log2’} |
|  | criterion | {‘mse’, ‘mae’} |
| *when used for classification* | *criterion* | *{‘gini’, ‘entropy’}* |
|  | *class_weight* | *{None, ‘balanced’, ‘balanced_subsample’}* |
