# Supplementary material for: Studying missingness in spinal cord injury data: challenges and impact of data imputation
Source: BMC Med Res Methodol. 2024 Jan 6;24:5. doi: 10.1186/s12874-023-02125-x (PMC10770973; doi:10.1186/s12874-023-02125-x)

**Additional File 2.** Comparison between pool-fit and fit-pool strategies in multiple imputation.

### Similarly as exposed in Section *Comparison of beta coefficients after linear regression (LR) using imputed data*, we compared the beta coefficients for the explanatory variables estimated from a LR, either pooling the imputed values into one common value first and fitting the LR using this unique imputed value (pool-fit), or fitting the LR on all imputed values before pooling the estimates into one common estimate (fit-pool).

As shown in the figure below, the two strategies lead to consistently different estimates when using random forest or pmm. However, the bias introduced would be negligible when compared to the bias introduced by the imputation itself (see **Figure 3**). Importantly, differences in fit-pool versus pool-fit approaches when imputing missing data with norm.predict were constantly close to zero, confirming it has the most effective imputation method in the simulation study presented.


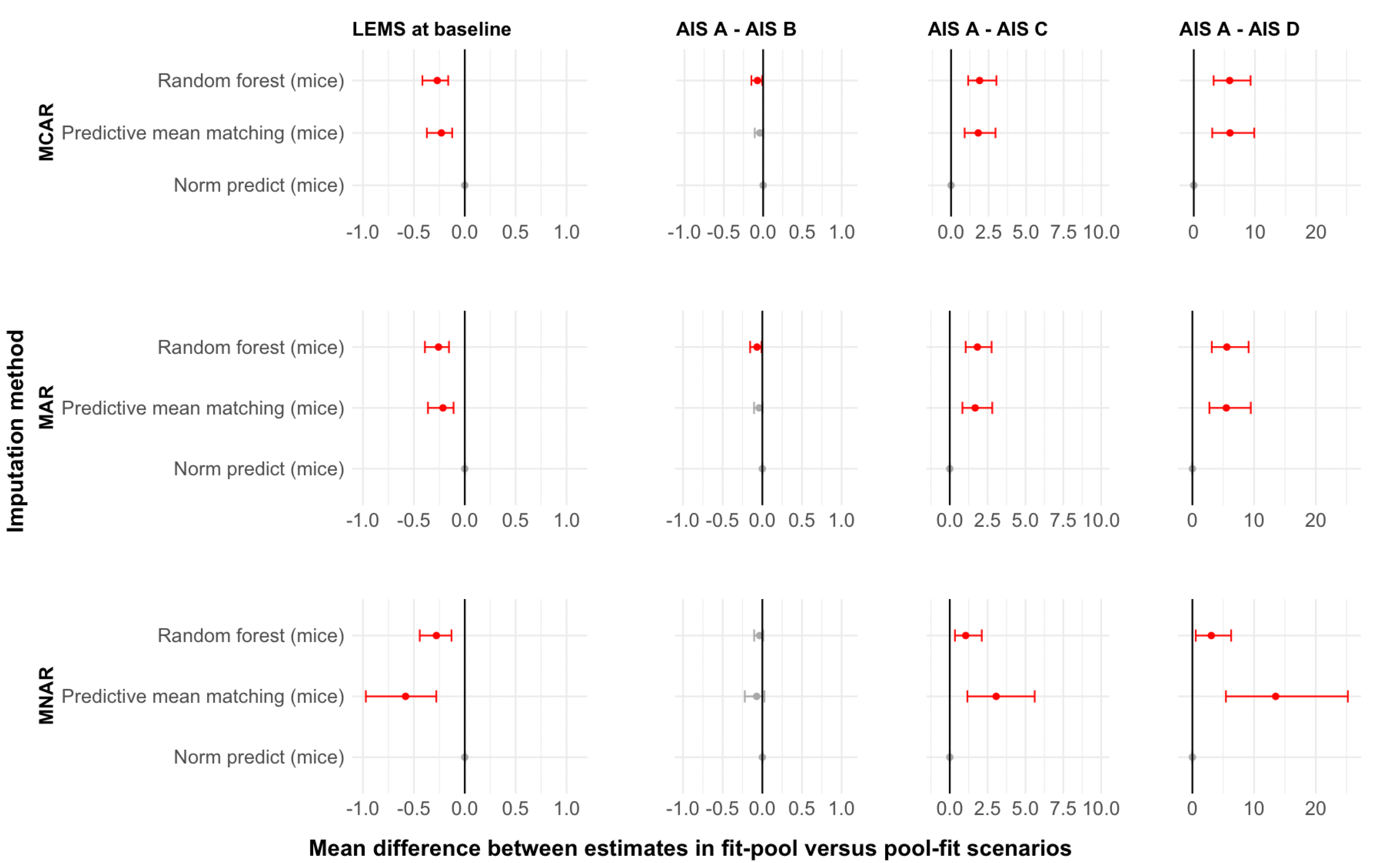

Supplement: Supplementary file 2 — Additional file 2. [file 12874_2023_2125_MOESM2_ESM.docx]
