# Supplementary material for: Studying missingness in spinal cord injury data: challenges and impact of data imputation
Source: BMC Med Res Methodol. 2024 Jan 6;24:5. doi: 10.1186/s12874-023-02125-x (PMC10770973; doi:10.1186/s12874-023-02125-x)
Supplement: Supplementary file 3 — Additional file 3. [file 12874_2023_2125_MOESM3_ESM.docx]

**Additional File 3.** Number of subsets for which the null hypothesis of the Little’s test is rejected, with a threshold of 0.05, for different distributions of AIS grades. Note that missing data was considered according to one pattern and in one variable at a time. MCAR: missing completely at random, MAR: missing at random, MNAR: missing not at random, AIS: American Spinal Injury Association Impairment Scale, LEMS: lower extremity motor score

| Little’s test | Outcome at week 52 | Sygen complete cases (subsets) | Balanced (subsets) |
| --- | --- | --- | --- |
| LEMS at week 01 | MCAR | 27 | 29 |
|  | MAR | 500 | 500 |
|  | MNAR | 500 | 500 |
| LEMS at week 52 | MCAR | 26 | 32 |
|  | MAR | 500 | 500 |
|  | MNAR | 500 | 491 |
