# Supplementary material for: Studying missingness in spinal cord injury data: challenges and impact of data imputation
Source: BMC Med Res Methodol. 2024 Jan 6;24:5. doi: 10.1186/s12874-023-02125-x (PMC10770973; doi:10.1186/s12874-023-02125-x)
Supplement: Supplementary file 6 — Additional file 6. [file 12874_2023_2125_MOESM6_ESM.docx]

**Additional File 6**. Number of subsets for which the null hypothesis of the two-sample Kolmogorov-Smirnov test is rejected. The test compares the distribution of lower extremity motor score at recovery in the baseline cohort (all values known) compared to the same variable after missing values were introduced and further imputed. Note that missing data was considered according to one pattern and in one variable at a time. MCAR: missing completely at random, MAR: missing at random, MNAR: missing not at random, LOCF: last observation carried forward, SVM: support vector machines, RBF: radial basis function

| Missingness pattern | Imputation method | Sygen complete cases (subsets) | Balanced (subsets) |
| --- | --- | --- | --- |
| MCAR | Mean | 500 | 500 |
|  | LOCF | 0 | 0 |
|  | Linear regression | 500 | 418 |
|  | K-nearest neighbors | 279 | 143 |
|  | Random forest | 175 | 156 |
|  | SVM with linear kernel | 500 | 313 |
|  | SVM with RBF kernel | 500 | 137 |
|  | Norm predict (mice) | 500 | 249 |
|  | Predictive mean matching (mice) | 500 | 412 |
|  | Random forest (mice) | 500 | 496 |
| MAR | Mean | 500 | 500 |
|  | LOCF | 0 | 0 |
|  | Linear regression | 500 | 440 |
|  | K-nearest neighbors | 280 | 148 |
|  | Random forest | 170 | 180 |
|  | SVM with linear kernel | 500 | 309 |
|  | SVM with RBF kernel | 500 | 151 |
|  | Norm predict (mice) | 500 | 277 |
|  | Predictive mean matching (mice) | 500 | 433 |
|  | Random forest (mice) | 500 | 496 |
| MNAR | Mean | 500 | 500 |
|  | LOCF | 0 | 0 |
|  | Linear regression | 276 | 368 |
|  | K-nearest neighbors | 65 | 46 |
|  | Random forest | 35 | 44 |
|  | SVM with linear kernel | 345 | 480 |
|  | SVM with RBF kernel | 95 | 26 |
|  | Norm predict (mice) | 131 | 132 |
|  | Predictive mean matching (mice) | 40 | 67 |
|  | Random forest (mice) | 461 | 327 |
