# Supplementary figures and images for: Studying missingness in spinal cord injury data: challenges and impact of data imputation
Source: BMC Med Res Methodol. 2024 Jan 6;24:5. doi: 10.1186/s12874-023-02125-x (PMC10770973; doi:10.1186/s12874-023-02125-x)

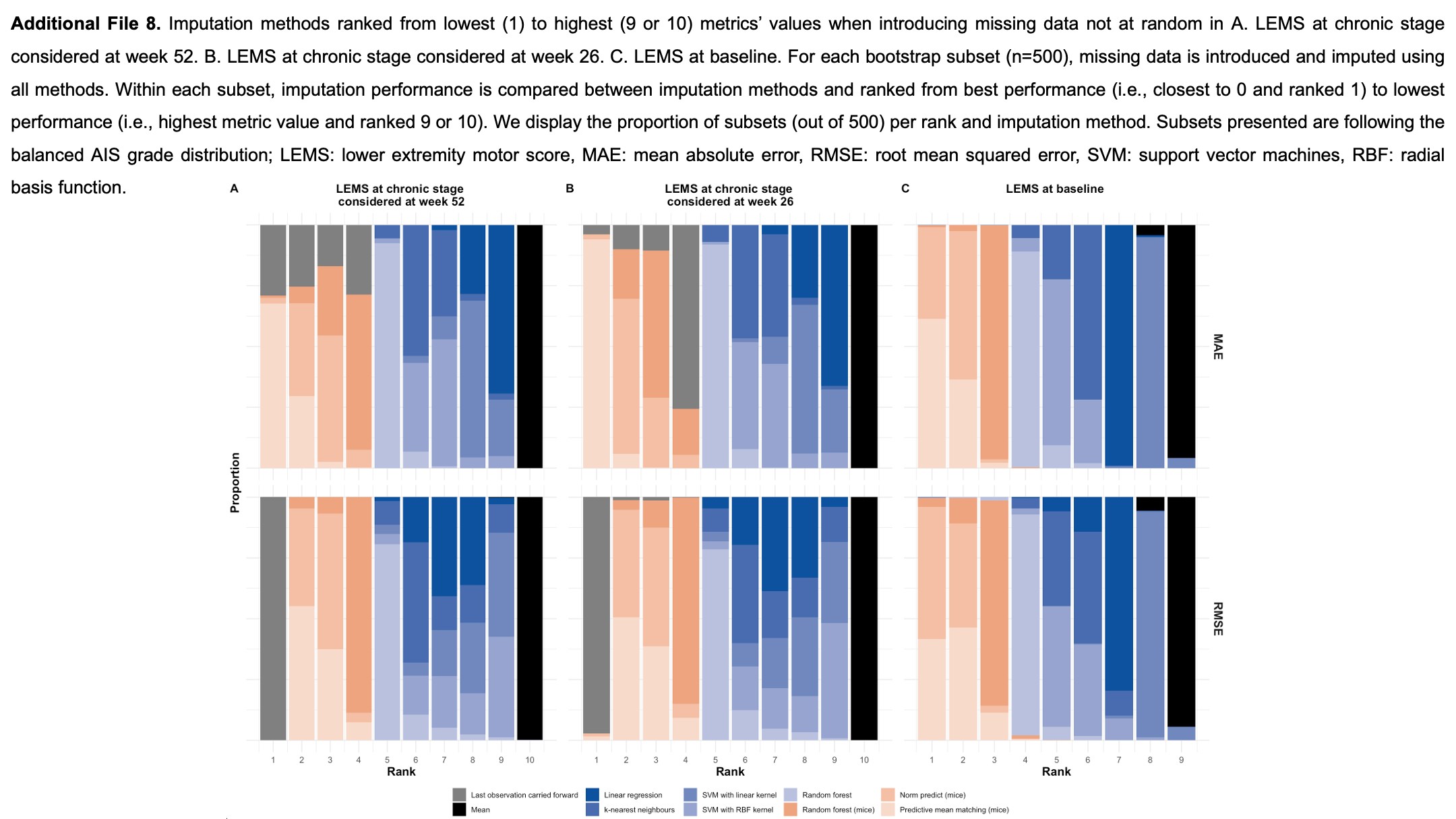

Supplement: Supplementary file 8 — Additional file 8. [file 12874_2023_2125_MOESM8_ESM.png]

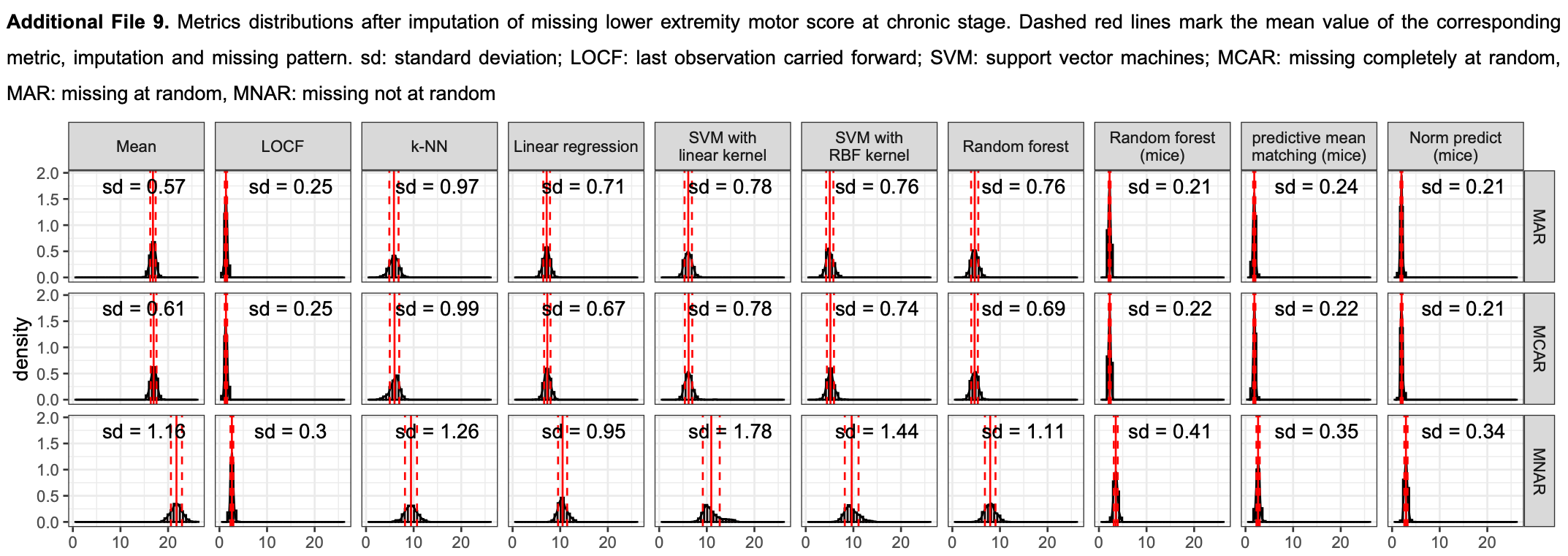

Supplement: Supplementary file 9 — Additional file 9. [file 12874_2023_2125_MOESM9_ESM.png]

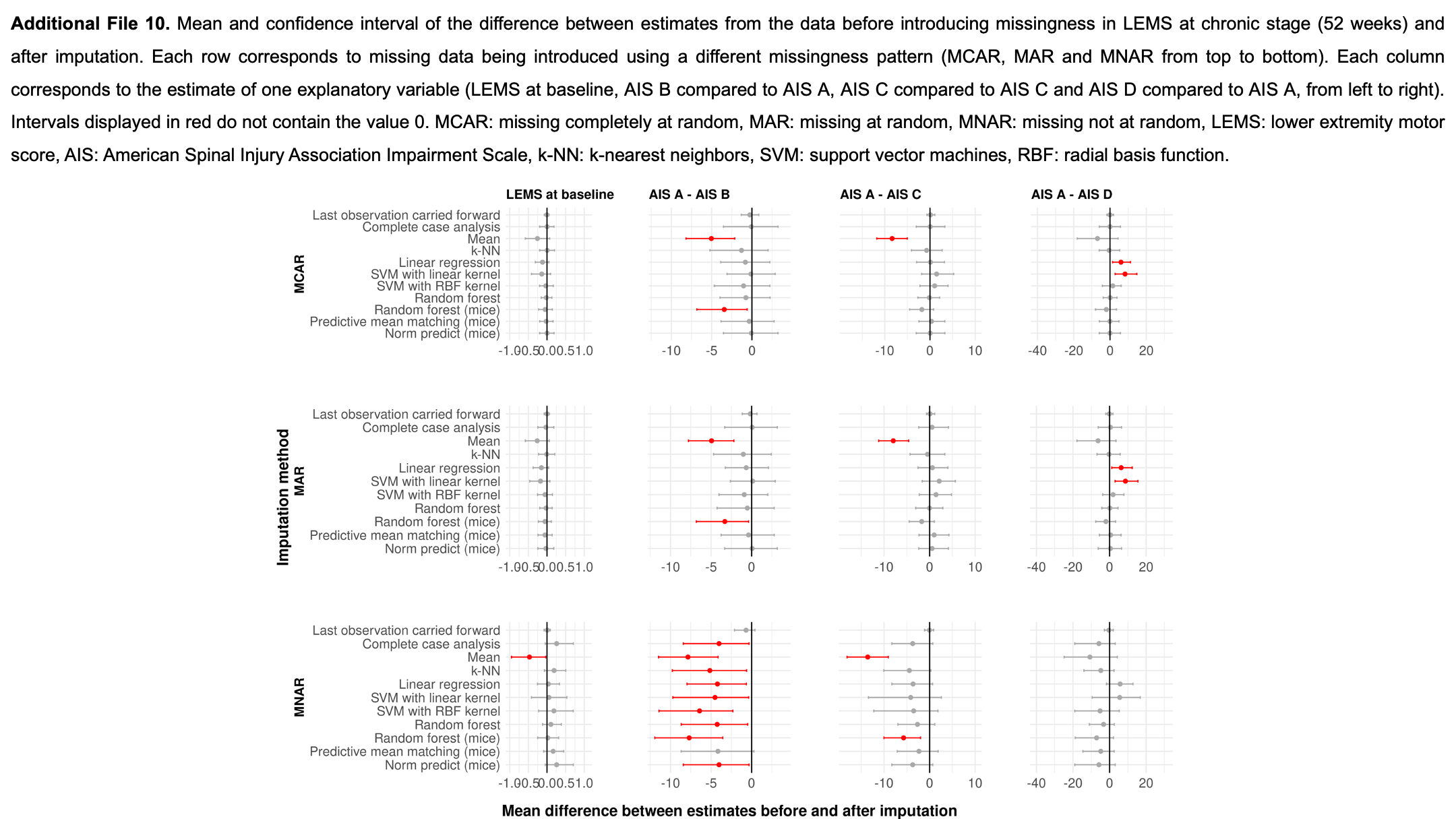

Supplement: Supplementary file 10 — Additional file 10. [file 12874_2023_2125_MOESM10_ESM.png]

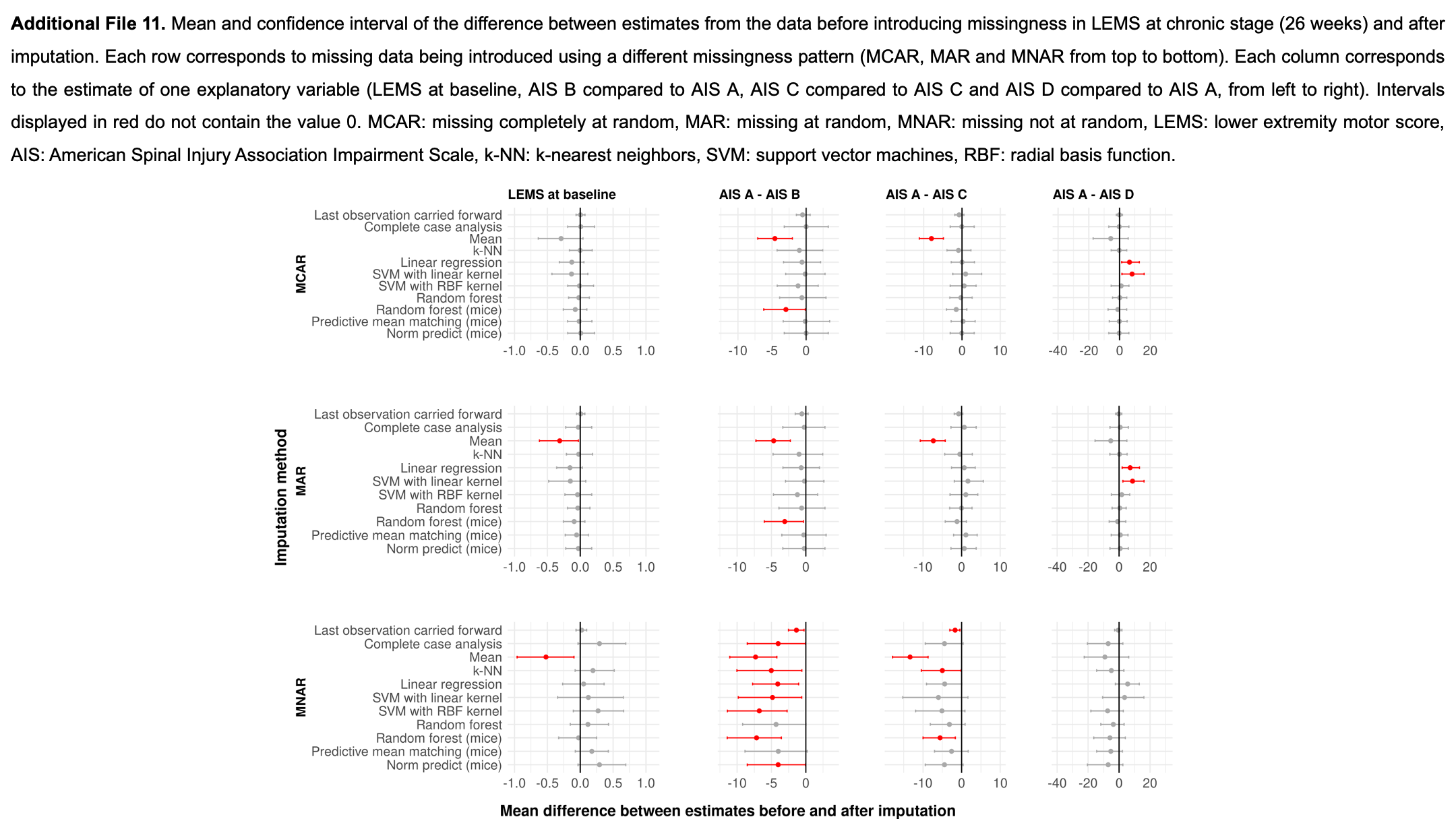

Supplement: Supplementary file 11 — Additional file 11. [file 12874_2023_2125_MOESM11_ESM.png]

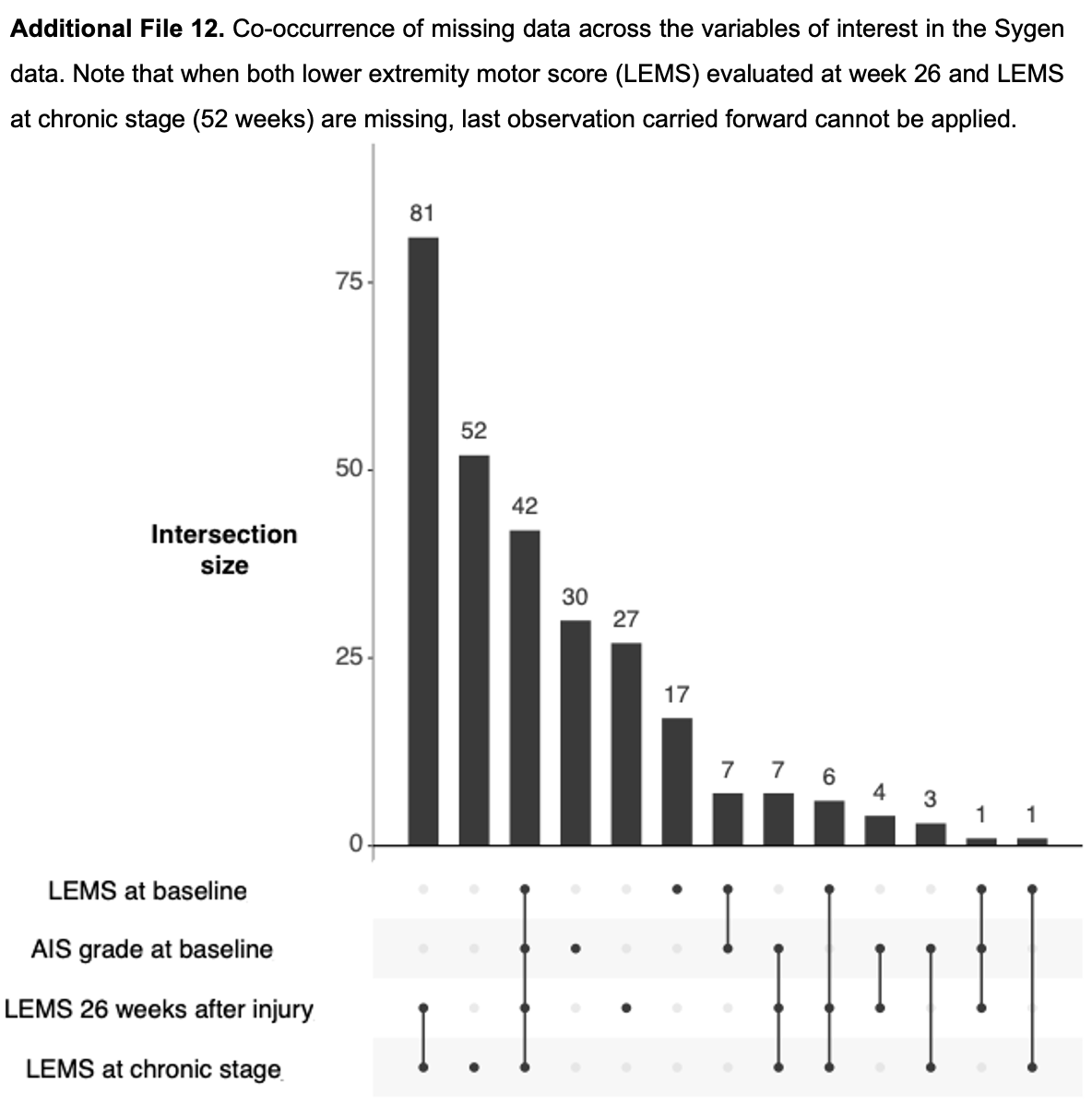

Supplement: Supplementary file 12 — Additional file 12. [file 12874_2023_2125_MOESM12_ESM.png]
